# Supplementary material for: Suppression of hollow droplet rebound on super-repellent surfaces
Source: Nat Commun. 2023 Sep 4;14:5386. doi: 10.1038/s41467-023-40941-3 (PMC10477213; doi:10.1038/s41467-023-40941-3)
Supplement: Supplementary file 1 — Supplementary Information [file 41467_2023_40941_MOESM1_ESM.pdf]

Supplementary information for

Suppression of hollow droplet rebound on super-repellent surfaces

Ying Zhou[1#], Chenguang Zhang[2#], Wenchang Zhao[1], Shiyu Wang[1], Pingan Zhu[1,3]*

[1]Department of Mechanical Engineering, City University of Hong Kong, Hong Kong 999077, China

[2]Ansys Inc., 10 Cavendish Ct, Lebanon, NH 03766, USA

[3]Shenzhen Research Institute, City University of Hong Kong, Shenzhen 518057, China.

[#]These authors contributed equally: Ying Zhou, Chenguang Zhang.

*e-mail: pingazhu@cityu.edu.hk

File contents:

Supplementary Figs. 1 to 10

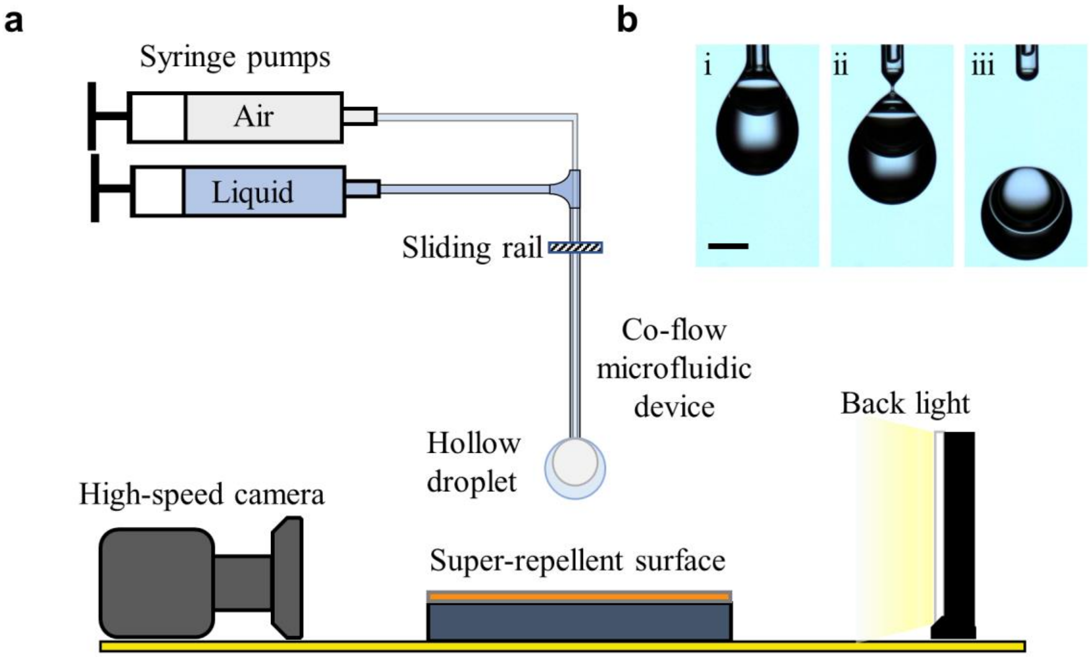

Supplementary Fig. 1 | Experimental setup. a Schematic of the experimental setup. **b** Snapshots showing the growing (i), detaching (ii), and falling (iii) of the HD. Scale bar, 1 mm.

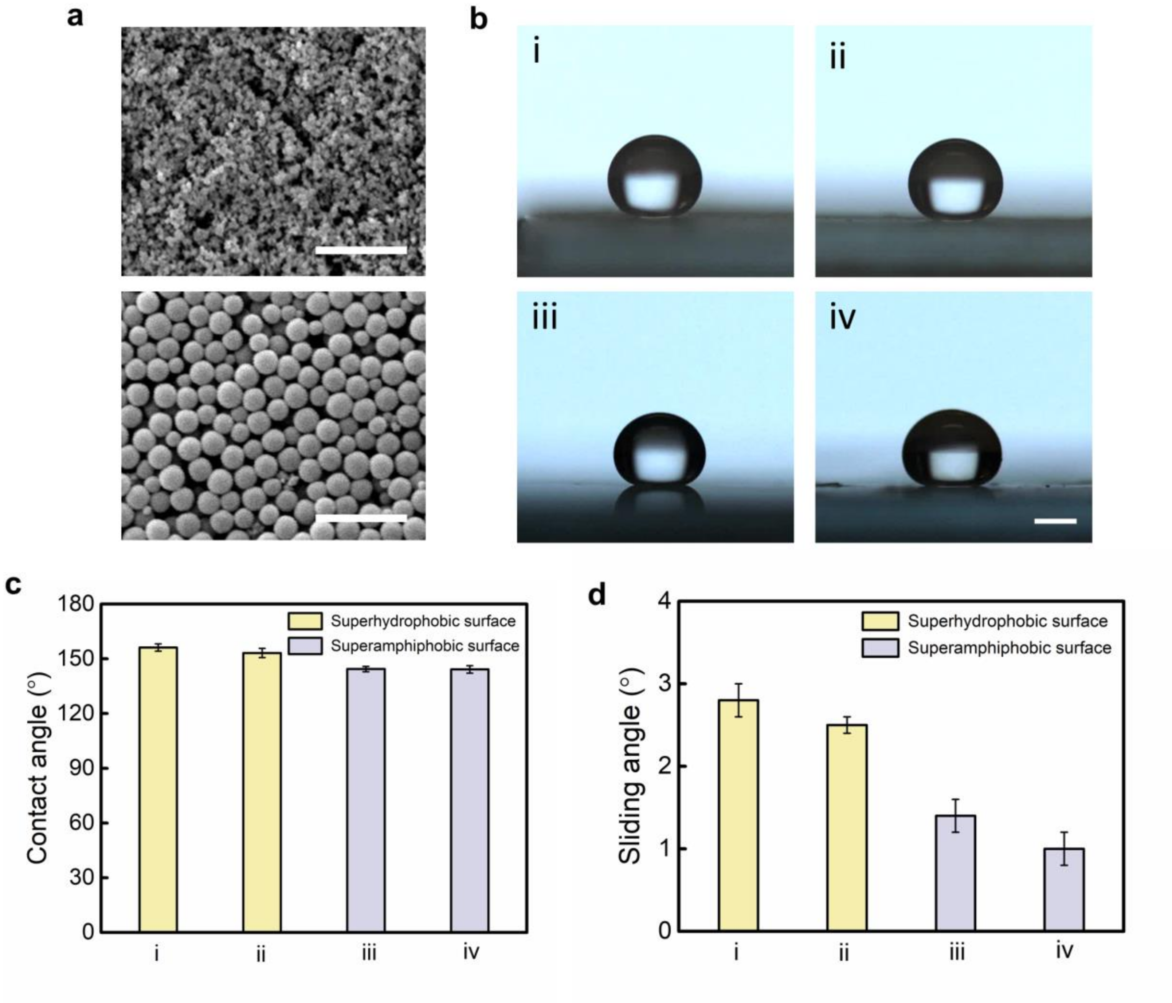

Supplementary Fig. 2 | Characterization of surface super-repellency. a Scanning electron microscope (SEM) images of the superhydrophobic (upper) and superamphiphobic (lower) surfaces. Scale bar, 1 μm. **b** Liquid drops beading up on super-repellent surfaces. The volume of droplets is 7.5 μL. (i) A pure water drop on the superhydrophobic surface; (ii) a 0.003 wt% SDBS drop on the superhydrophobic surface; (iii) a 0.2 wt% SDBS drop on the superamphiphobic surface; (iv) a *n*-hexadecane drop on the superamphiphobic surface. Scale bar, 1 mm. **c** and **d** Contact angle (**c**) and sliding angle (**d**) of liquid droplets on super-repellent surfaces. Groups (i) to (iv) correspond to cases (i) to (iv) in (**b**), respectively. Error bars in (**c**) and (**d**) were obtained from the standard deviation of three replicate experiments.

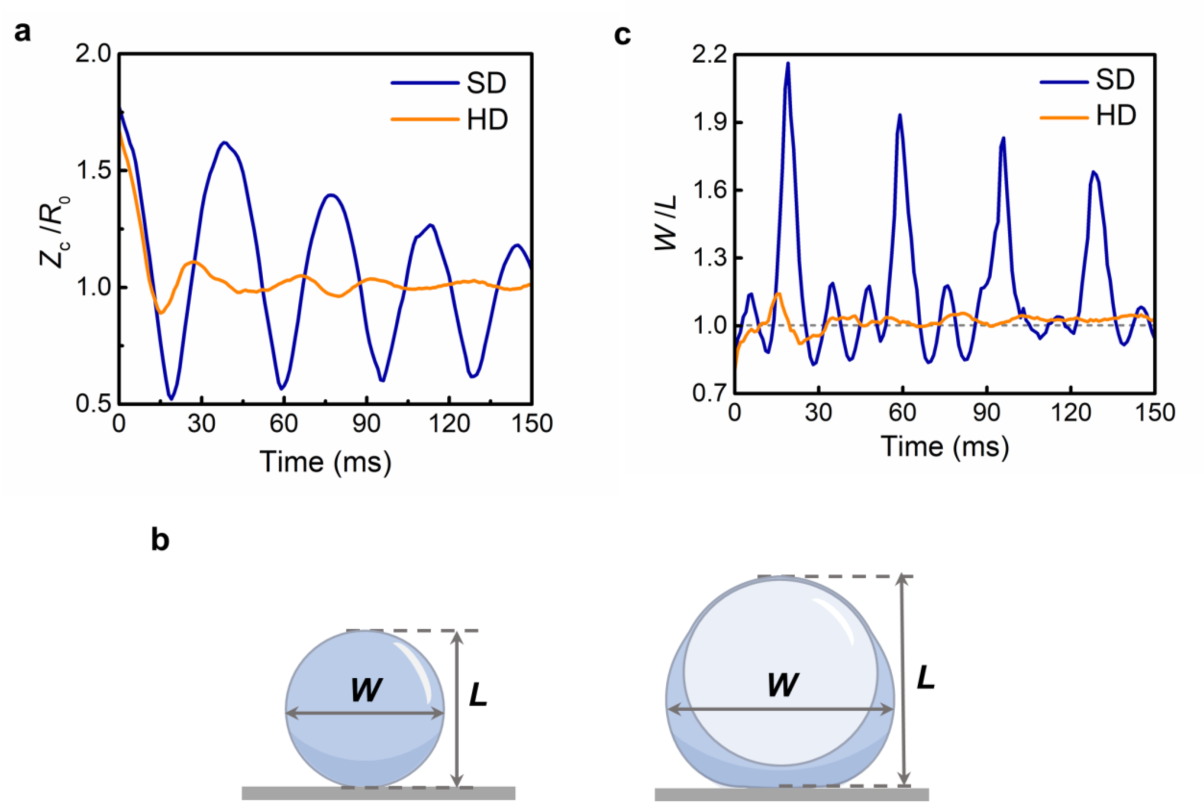

Supplementary Fig. 3 | Shape deformation of the impacting SD and HD. a Dependence of the centroid position (Z_c) of droplets on time. **b** Schematic displaying the width (*W*) and longitudinal height (*L*) of the SD (left) and the HD (right). **c** Dependence of the aspect ratio (*W*/*L*) of droplets on time. The SD bounces off while the HD rests on the super-repellent surface after impact.

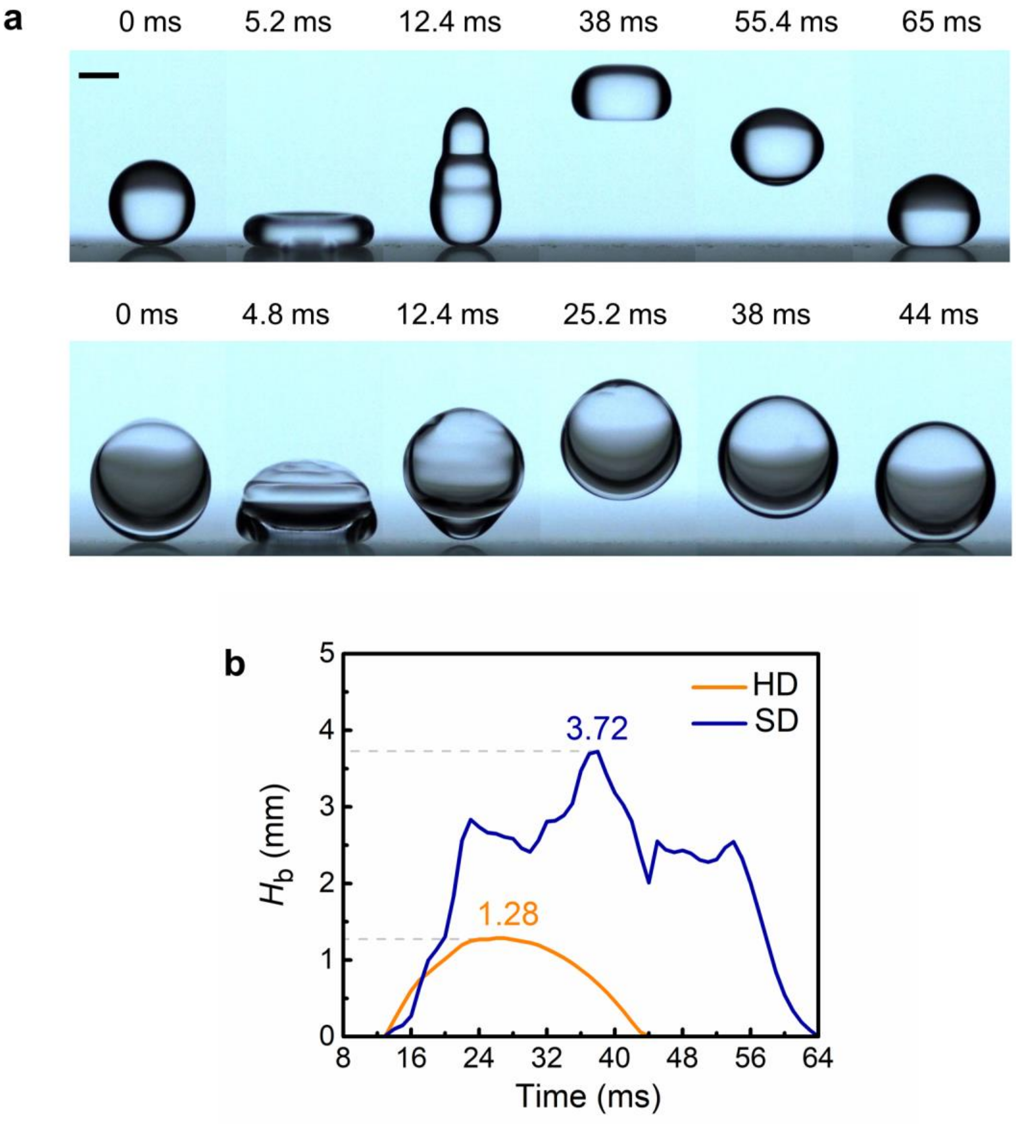

Supplementary Fig. 4 | Rebound of impacting droplets at high *We*. a Snapshots showing the rebound of SD (upper row) and HD (lower row) impacting a superhydrophobic surface when released at H_0 = 10 mm. The HD quickly restores the spherical shape while the SD vibrates agitatedly after taking off. The liquid surface tension is 53 mN m^{-1}. **b** Comparison of the bottom height H_b between the SD and HD after taking off. The maximum rebound height of HD is nearly three times lower than that of SD, indicating the significantly reduced restitution coefficient of the bouncing HD. Scale bar, 1 mm.

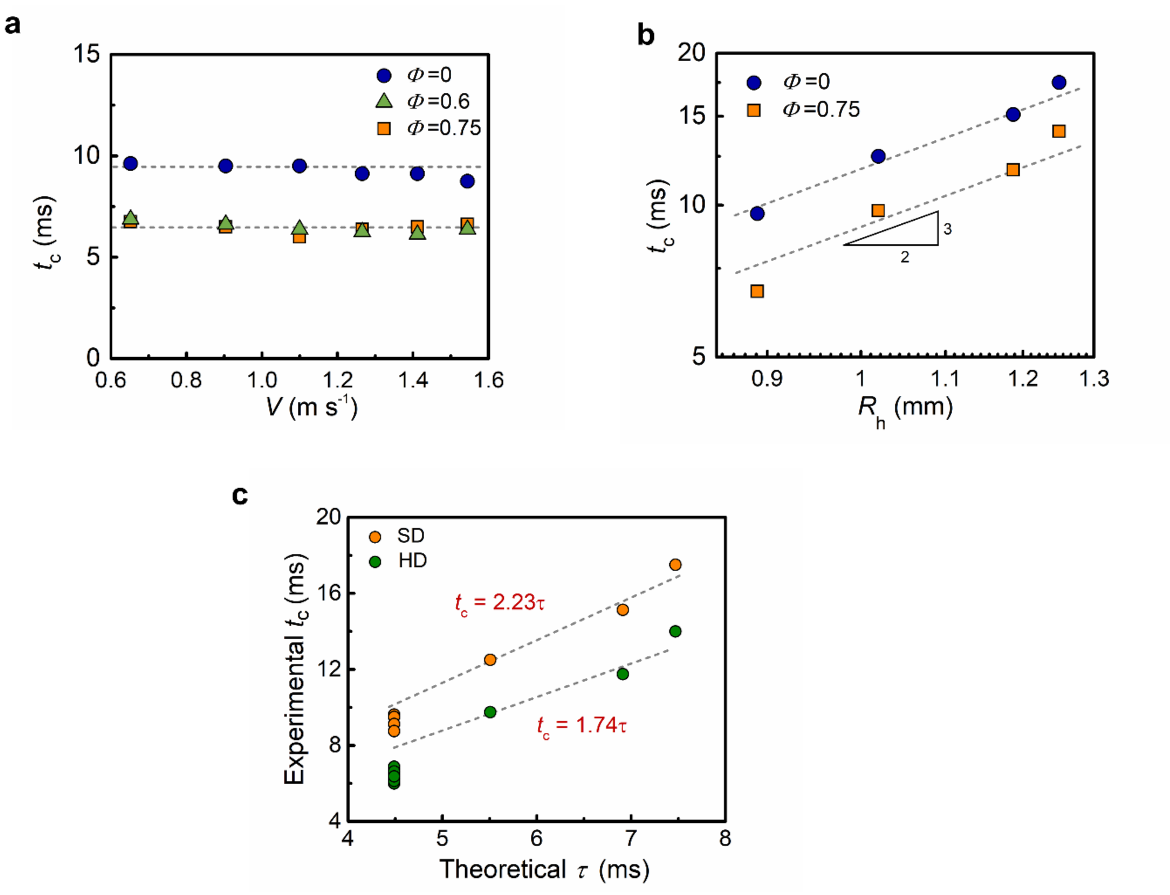

Supplementary Fig. 5 | Contact time of impacting droplets. a The contact time as a function of impact velocity V_0 for different Φ. R_h = 0.89 mm. **b** The contact time against the apparent radius R_h. V_0 = 0.65 m s^{-1}. **c** Camparision of the experimental contact time t_c against theoretical inertial-capillary timescale τ. Data was obtained by using droplets (35 mN m^{-1}) to impact the superamphiphobic surface.

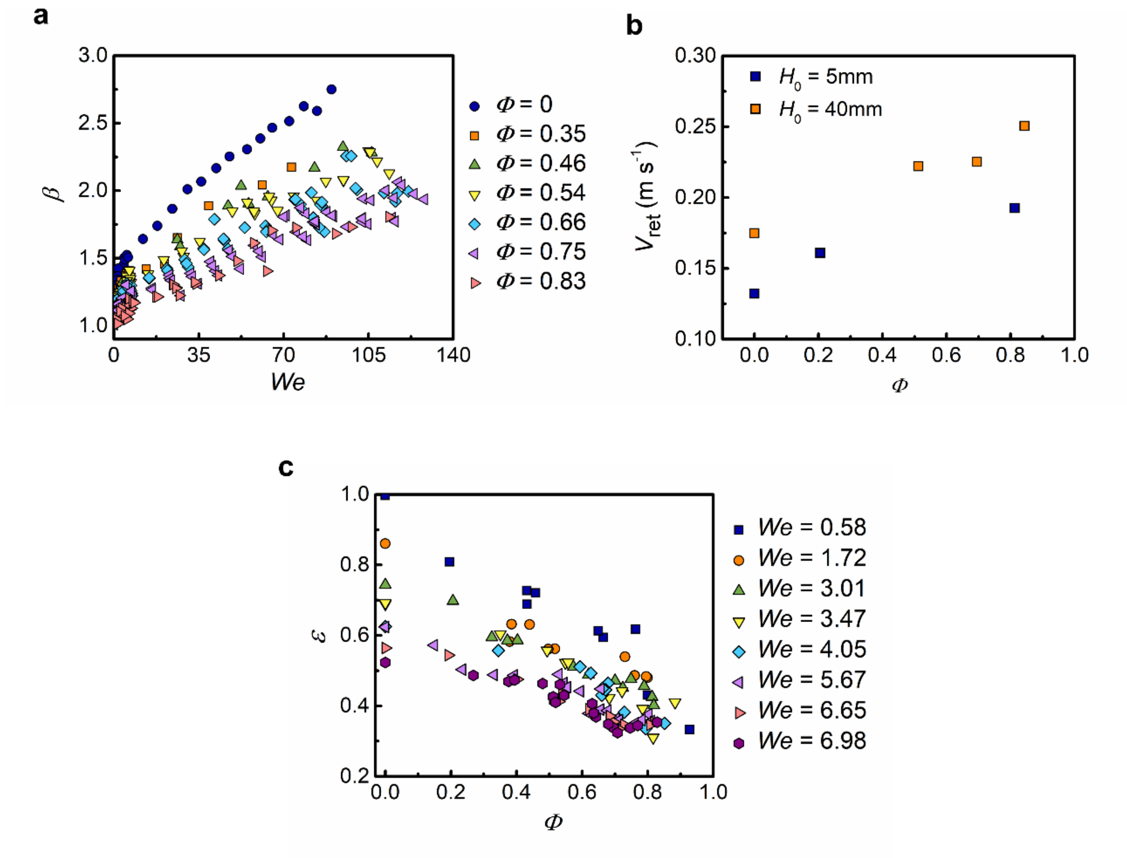

Supplementary Fig. 6 | Bouncing dynamics of impacting droplets. a The change of the spreading factor β with different volume fraction Φ and Weber number *We*. **b** The change of the retraction velocity V_{ret} with different volume fraction Φ and impact height H_0. **c**, The change of the restitution coefficient ε with different volume fraction Φ and Weber number *We*.

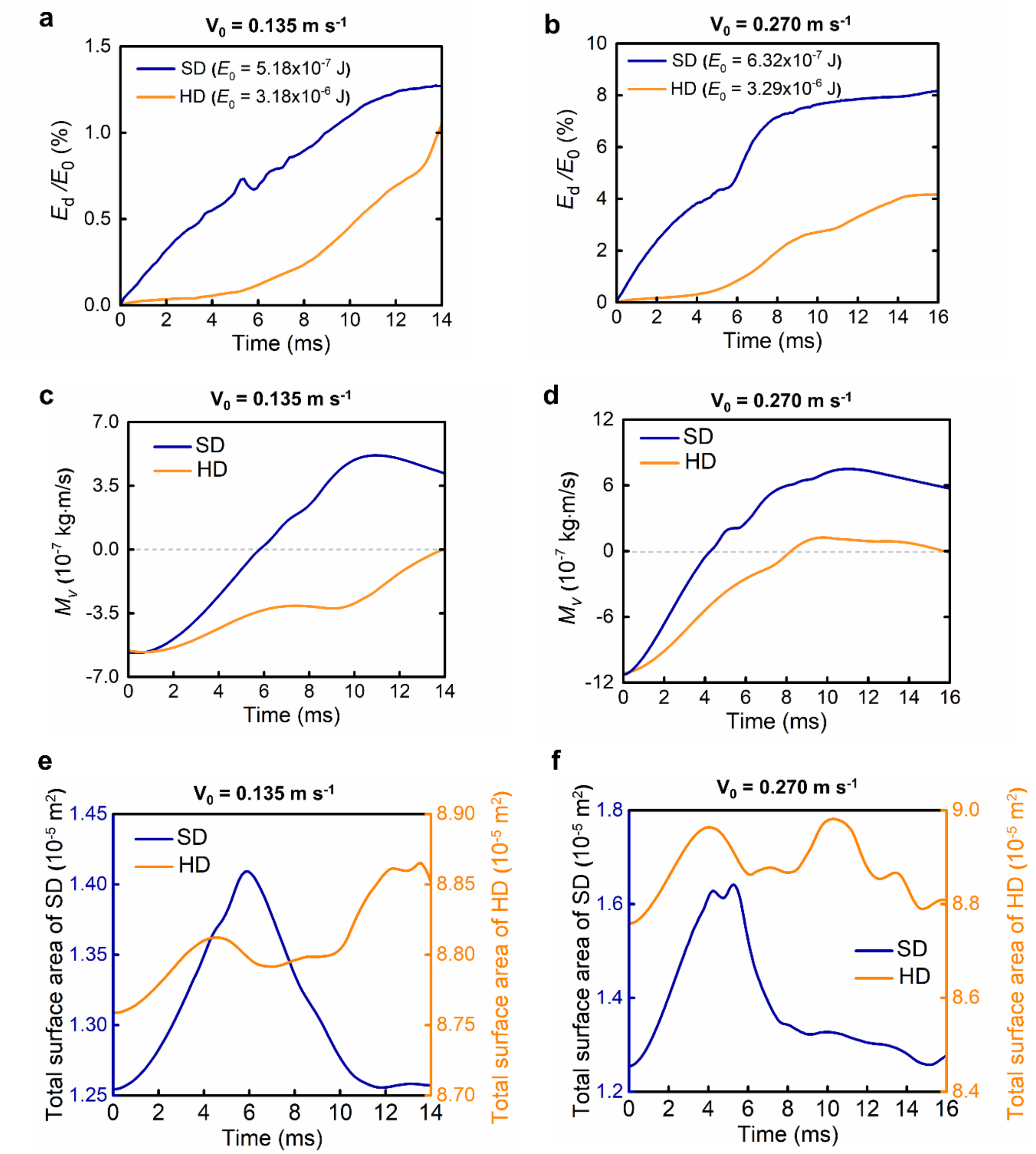

Supplementary Fig. 7 | Numerical simulation of impacting SD and HD. a and **b** The ratio of energy dissipation (E_d) to the total energy (E_0) of impacting droplets at the impact velocity of 0.135 m s^{-1} (**a**) and 0.270 m s^{-1} (**b**). **c** and **d** The change of out-of-plane momentum (M_v) with time at the impact velocity of 0.135 m s^{-1} (**c**) and 0.270 m s^{-1} (**d**). **e** and **f** Contrast in the total surface area between SD and HD at the impact velocity of 0.135 m s^{-1} (**e**) and 0.270 m s^{-1} (**f**).

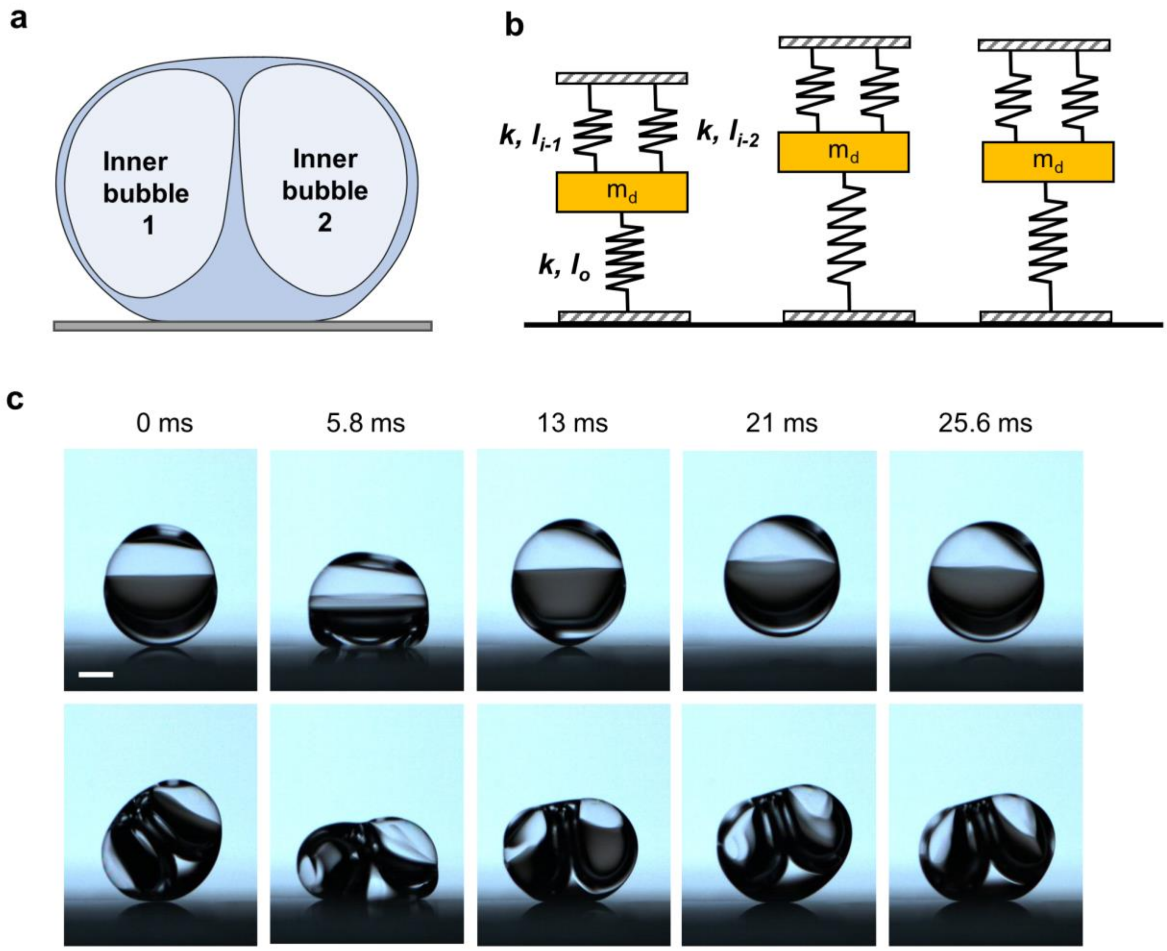

Supplementary Fig. 8 | Contrast between single-bubble and double-bubble HD. a Schematic showing the HD encapsulating two bubbles. **b** The mass-spring model for the double-bubble HD, where the two bubbles are modelled by two parallel springs with a length of $l_{i\text{-}1}$ and $l_{i\text{-}2}$, respectively. **c** Snapshots displaying the bouncing dynamics of the single-bubble (upper row) and double-bubble (lower row) HD after impingement. The two droplets have the same bubble volume fraction (Φ = 0.83) and mass (m = 9 mg). When released from the same height, the single-bubble HD bounces, whereas the double-bubble HD rests on the super-repellent surface without rebound, demonstrating the enhanced counteractive capillary effects. Snapshots were obtained by using droplets (35 mN m^{-1}) to impact the superamphiphobic surface. Scale bar, 1 mm.

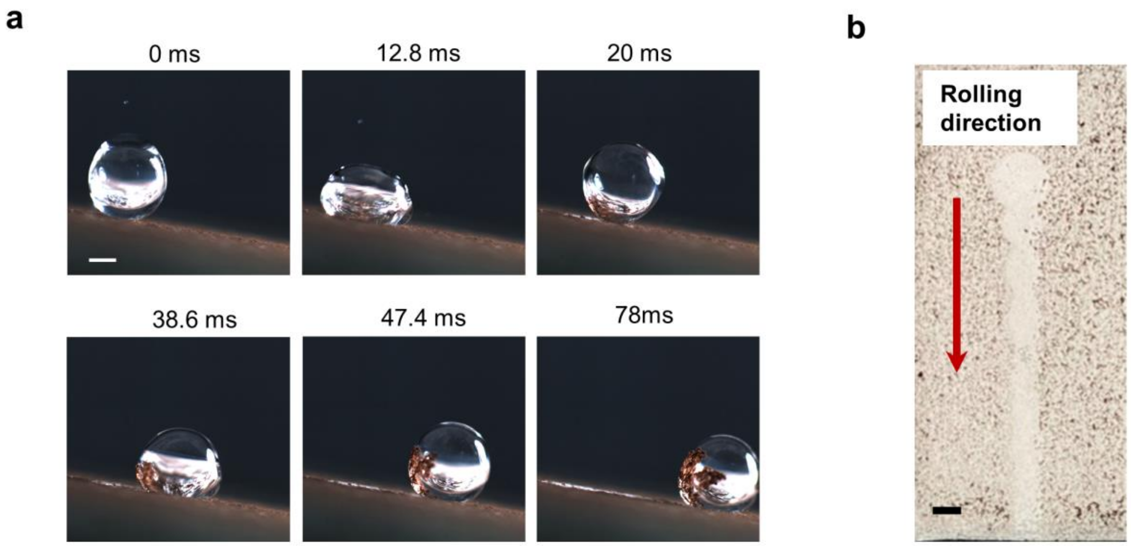

Supplementary Fig. 9 | Self-cleaning by HD impact. a Snapshots displaying the rolling behaviour of the HD impacting an inclined superamphiphobic surface covered with dust particles. **b** A picture showing the result of self-cleaning by the rolling HD. Scale bars, 1 mm.

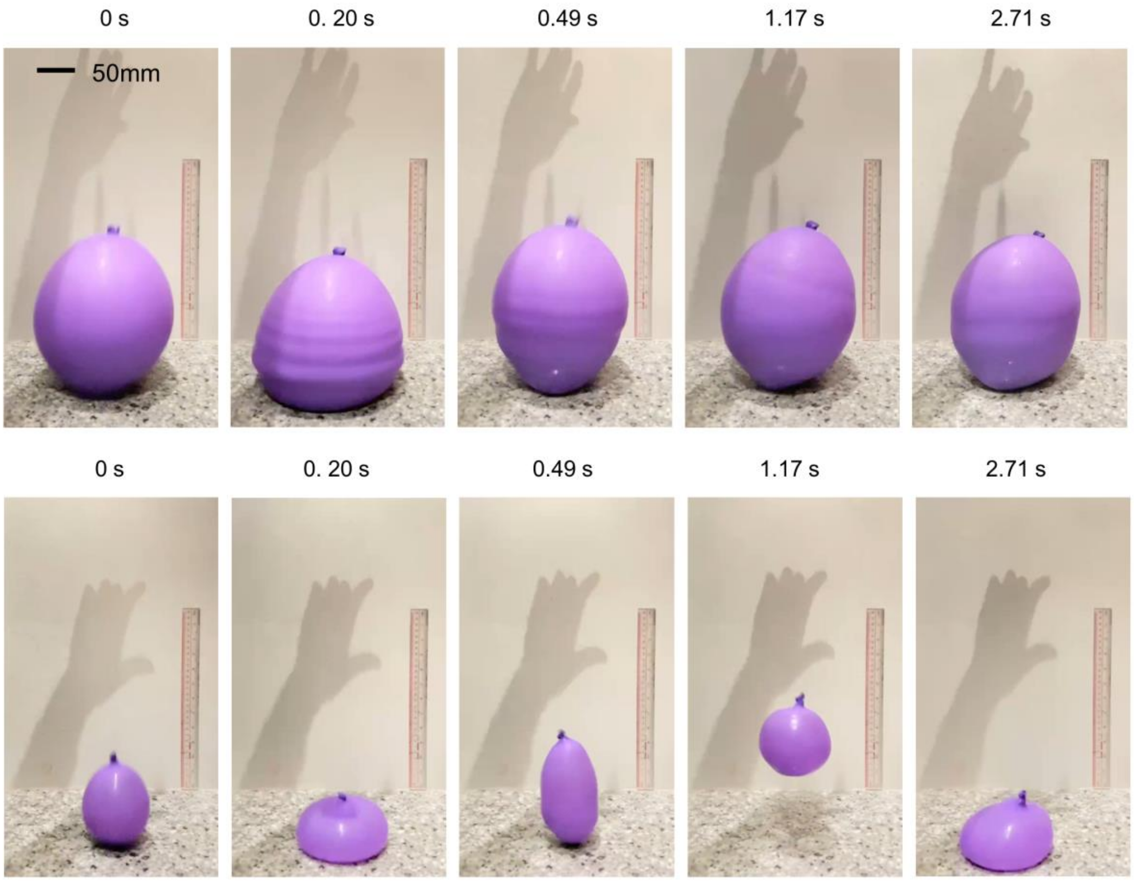

Supplementary Fig. 10 | A macroscopic shock-absorbing hollow system. A gas-core/water-shell compound balloon rests on the ground, notably contrasting the rebound of the single-phase water-filled balloon. As such, behaviours of the compound and sing-phase balloons imitate those of HDs and SDs, respectively, which convincingly extends the droplet-based shock absorber into the macroscopic realm. The length of the ruler is 35 cm.
