# Supplementary material for: Suppression of hollow droplet rebound on super-repellent surfaces
Source: Nat Commun. 2023 Sep 4;14:5386. doi: 10.1038/s41467-023-40941-3 (PMC10477213; doi:10.1038/s41467-023-40941-3)
Supplement: Supplementary file 3 — Description of Additional Supplementary Files [file 41467_2023_40941_MOESM3_ESM.pdf]

Description of Additional Supplementary Files

Supplementary Movie 1: Bouncing dynamics of SD and HD on a superhydrophobic surface. The two droplets have the same characteristic radius R_h = 1.08 mm, and the same release height H_0 = 0.9 mm. The bubble volume fraction of HD is Φ = 0.82. After impact, the SD completely bounces off while the HD rests on the surface without a rebound.

Supplementary Movie 2: Universal non-rebound of HD on super-repellent surfaces. Six proof-of-concept cases are shown: (i) the air-in-hexadecane HD impacting a superamphiphobic surface; (ii) the air-in-pure water HD impacting a superhydrophobic surface; the air-in-SDBS HD impacting the horizontal (iii) and 10°-tilted (iv) surfaces in Leidenfrost regime; (v) the air-in-SDBS HD impacting a vertical superamphiphobic surface; (vi) the air-in hexadecane HD impacting a solid surface under-liquid. In case (i), the HD with Φ = 0.9 was released at H_0 = 0.4 mm; in case (ii), the HD with Φ = 0.75 was released at H_0 = 0.37 mm; for (iii) and (iv), HDs with Φ = 0.94 were released at H_0 = 0.15 and 0.3 mm, respectively; in case (v), the HD of Φ = 0.84 was set to impact the surface with a velocity of 0.08 m s^{-1}; in case (vi), the HD of Φ = 0.7 was released under an aqueous solution of 1% polyvinyl alcohol (PVA), ascending and impacting the glass plate driven by buoyancy. The surface temperature was 210 ± 5°C, and the concentration of SDBS was 0.1 wt% in Leidenfrost experiments. The impacts of SD under the same experimental conditions were used for comparison. In each case, SD bounced off while HD did not.

Supplementary Movie 3: Rebound of droplets at higher Weber numbers. The video contrasts the rebound of SD and HD (Φ = 0.78) after impacting a superhydrophobic surface when released at H0 = 10 mm. Although both droplets rebound after impact, HD is less lively in comparison with SD. For example, the shape of HD is much less vibrant, and the bouncing height is largely reduced.

Supplementary Movie 4: : Numerical simulation of droplet impact at different impact velocities. The video shows the shape change and internal flow of SDs and HDs at an impact velocity of V0 = 0.135 m s –1 (i) and V0 = 0.270 m s–1 (ii). Both droplets have the same characteristic radius Rh = 1 mm, liquid density ρ = 1000 kg m–3 , and surface tension γ = 35 mN m–1 . The bubble volume fraction of the HDs is Φ = 85.7%. The contact angle of the liquid on the super-repellent surface is set at 180°to avoid any solid-liquid adhesion-induced energy dissipation.

Supplementary Movie 5: Enhancing rebound suppression by encapsulating multiple bubbles. The video contrasts the impact of a single-bubble and a double-bubble HD on a superamphiphobic surface. The two HDs have the same bubble volume fraction Φ = 0.83 and only differ from each other in the number of encapsulated bubbles. When released at the same height H0 = 1.4 mm, the singlebubble HD bounces off while the double-bubble HD stays on the superamphiphobic surface.

Supplementary Movie 6: : Surface self-cleaning by HD impact. A superamphiphobic surface is coated with $Fe3O4$ nanoparticles as the modeled dust. Released at H0 = 3 mm, the HD (Φ =0.72) impacts the 10°-tilted superhydrophobic surface and rolls down, which absorbs dust particles for surface cleaning.

Supplementary Movie 7: Non-rebound of macroscopic hollow systems. A gas-core/water-shell compound balloon and a water-filled balloon are released at H0 = 250 mm to impact the ground. The waterfilled balloon rebounds while the compound balloon stays on the ground by absorbing the shock, similar to the impact of SD and HD on super-repellent surfaces, respectively
